# Total Knee Arthroplasty in Haemophilia: Long-Term Results and Survival Rate of a Modern Knee Implant with an Oxidized Zirconium Femoral Component

**DOI:** 10.3390/jcm12134356

**Published:** 2023-06-28

**Authors:** Christian Carulli, Matteo Innocenti, Rinaldo Tambasco, Alessandro Perrone, Roberto Civinini

**Affiliations:** Orthopaedic Clinic, University of Florence, 50139 Florence, Italy; christian.carulli@unifi.it (C.C.);

**Keywords:** haemophilia, knee arthroplasty, inhibitors, loosening, oxidized zirconium, long-term results

## Abstract

(1) Background: Total Knee Arthroplasty (TKA) in patient with haemophilia (PWH) has usually been performed with the use of cobalt-chrome femoral and titanium tibial components, coupled with standard polyethylene (PE) inserts. The aim of this retrospective study was to evaluate the long-term outcomes and survival rates of TKA in a series of consecutive PWH affected by severe knee arthropathy at a single institution. (2) Methods: We followed 65 patients undergoing 91 TKA, implanted using the same implant, characterized by an oxidized zirconium femoral component, coupled with a titanium tibial component, and a highly crosslinked PE. At 1, 6, and 12 months; then every year for 5 years; and finally, every other 3 years, all patients were scored for pain (VAS), function (HJHS; KSS), ROM, and radiographic changes. Kaplan–Meier survivorship curves were used to calculate the implant survival rates. (3) Results: The mean follow-up was 12.3 years (4.2–20.6). All clinical and functional scores improved significantly from preoperatively to the latest follow-up (VAS: from 6.9 to 1.3; HJHS: from 13.4 to 1.9; KSS: from 19.4 to 79; ROM: from 42.4° to 83.6°). The overall survivorship of the implants was 97.5% at the latest follow-up. (4) Conclusions: The present series showed a high survival rate of specific implants potentially linked to the choice of an oxidized zirconium coupled with a highly crosslinked PE. We promote the use of modern implants in these patients in order to ensure long-lasting positive outcomes.

## 1. Introduction

Haemophilia is one of the most frequent rare diseases, consisting of a congenital lack of specific coagulative factors VIII (FVIII, haemophilia A) or IX (FIX, haemophilia B) through an inherited X-linked recessive condition. Each of these clotting factors plays a role in the intrinsic pathway of blood coagulation [1,2]. The prevalence of haemophilia is commonly reported as 1 in 5000 in the male population and 1 in 10,000 overall [3]. The prevalence of haemophilia A is approximately 1 in 5000 male live births, and that of haemophilia B is about 1 in 30,000 male live births [2,4]. Patients with haemophilia can have mild, moderate, or severe types of the condition, defined by plasma factor levels of 6–40%, 1–5%, or less than 1%, respectively [5]. Subjects with factor plasma levels less than 1 IU/dL are classified as severe haemophiliacs, whereas those with factor levels between 1 and 5 IU/dL and more than 5 IU/dL are affected by moderate and mild haemophilia [4]. However, the bleeding phenotype may be rather heterogeneous [6,7]. Patients with haemophilia (PWH), almost exclusively males, suffer from frequent haemorrhages and hemarthroses from childhood [8]. In the past, this rare disease was associated with high rates of mortality in the case of “noble” organ bleedings, but in modern times and thanks to early preventive haematological management (periodic administration of recombinant coagulative factors), PWH mostly complain of joint pain and impairment in the so-called “target joints” (TJ). TJ are generally synovial joints (knees, elbows, ankles; less frequently hips and shoulders) that develop synovitis and progressive deterioration on the basis of blood persistency in the articular space [9]. The cartilage is progressively damaged by iron deposition, lysosomal enzymes, and pro-inflammatory cytokines produced by the inflamed synovium, which eventually leads to subarticular bone cyst formation. Repeated hemarthroses are responsible for the development of synovial hyperplasia and angiogenesis, with further bleeding occurring in the friable and thickened synovium. Joint bleeding stretches the joint capsule and ligaments and leads to joint instability, which is worsened because reduced joint motility from pain causes peri-articular muscle weakness. In more advanced stages, the joint is grossly damaged by cartilage loss and subchondral bone sclerosis, which further limits movement and leads to crepitus and deformity. Soft-tissue swelling and effusions are rare, and joint contracture occurs from muscle retraction and bone ankylosis, particularly if the muscles are weak. The level of pain varies and fluctuates but may be severe [10]. Blood, in fact, induces a direct degenerative action on synovium and cartilage and, consequently, an indirect involvement of all intra-articular structures (capsule, ligaments, meniscus, labrum). The result is an early and severe specific arthritis, named “haemophilic arthropathy,” that alters the physiological development of young subjects: the more the number of involved joints, the worst the quality of life of PWH from childhood. The haematological prophylaxis alone is not enough to prevent this joint disease, even if it is considered the most important strategy [11]. Association with other approaches is mandatory. Several conservative treatments have been reported during recent years with high rates of clinical success when indicated for early stages of arthropathy, namely muscle maintenance, braces, anti-inflammatory drugs (paracetamol, cox-1 inhibitors), intra-articular injections, and physical therapy [11,12,13,14,15,16]. For persistent synovitis, an arthroscopic treatment is often necessary, while for severe arthropathy, joint replacement is the gold standard [17,18,19,20]. However, even if joint replacement with total knee arthroplasty in PWH is an effective treatment, it is also a different procedure than TKA in patients with primary arthritis because the pathophysiology of both conditions is substantially different. The arthropathy in PWH is usually characterized by repeated intra-articular bleedings with intra-articular deposits of hemosiderin and iron, which leads to the upregulation of pro-inflammatory cytokines and, consequently, synovial hypertrophy. This process also happens after the implantation of a TKA, potentially leading to early aseptic loosening of the prosthesis components. Consequently, in patients with bleeding disorders, the results of TKA are expected to be inferior to those in patients without bleeding disorders [18,21,22,23]. Patients with haemophilia are at risk for complications following orthopaedic surgery for a number of reasons. The risk of bleeding may be increased because of inadequate coagulation factor replacement, the presence of coagulation factor inhibitors, and/or structural articular damage. A higher prevalence of comorbidities, such as human immunodeficiency virus (HIV) and hepatitis C virus (HCV) infection, may predispose patients with haemophilia to postoperative infection and delayed wound-healing. Indeed, previous studies have reported that patients with haemophilia who underwent orthopaedic surgery had high rates of postoperative bleeding (39%), infection (7%), and delayed wound-healing (2.2%) [24]. Historically, the results of this surgery in haemophilic patients have shown good rates of success despite the high risk of complications mostly related to intra- or postoperative bleedings (causing early septic or aseptic loosening) and coinfections (hepatitis, HIV) [18,21,22,23]. Indeed, the key to success in such complex patients is not just the surgery itself but also and foremost a multidisciplinary approach. It has already been reported in the literature that through a multidisciplinary approach, with appropriate pre-and postoperative management of bleeding, good clinical results and lower complication rates can be obtained. In particular, to achieve satisfactory mid/long-term clinical results, it is detrimental to treat PWH in dedicated haemophilia comprehensive care centres where modern haematological management can be employed. Nevertheless, in the majority of the dedicated haemophilia centres, joint replacements with total knee arthroplasty (TKA) have been performed using old-generation cemented or standard implants. Specifically, in most series reported in the literature, TKA in PWH has been performed using cobalt-chrome femoral and titanium tibial components, coupled with standard polyethylene (PE) inserts [25,26,27,28,29,30,31,32,33,34]. We believe that the use of more modern implants could enhance the already described beneficial action of modern haematological agents in order to obtain long-term implant survivorship in PWH.

The aim of this retrospective study is the evaluation of the long-term clinical outcomes and survival rate of TKA in a series of consecutive PWH affected by severe knee arthropathy at a single institution, performed using a single modern knee implant, which is characterized by an oxidized zirconium femoral component, coupled with a titanium tibial component, and a highly crosslinked PE.

## 2. Materials and Methods

**Figure 1:**
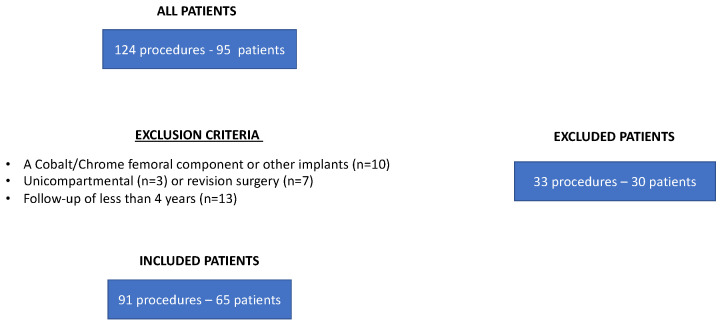
Cohort flow diagram of patient selection.

The medical records of all PWH undergoing a TKA at the authors’ institution in the period between 2001 and 2022 were evaluated, and a total of 124 procedures were found. The cohort flow diagram for patient selection (inclusion and exclusion) is shown in Figure 1. Inclusion criteria were subjects with Haemophilia A or B, having undergone a primary knee arthroplasty with a femoral component in oxidized zirconium, with a minimum follow-up of 4 years. Exclusion criteria were patients operated by a cobalt/chrome femoral component or other implants, patients operated by unicompartmental or revision surgery, patients with a follow-up of less than 4 years. The final study population consisted of 65 patients, undergoing a total of 91 TKA (52 right knees, 39 left knees; 13 bilateral TKA: 12 staged, 1 simultaneous), with a mean age at the time of surgery of 39.3 years (range: 23–64) and a mean BMI of 24.1 (range 21–29). All patients were male and were affected by Haemophilia A (52 patients) and B (13 patients). Overall, 20 patients had inhibitors (antibodies against recombinant factors). Four of them had a chronic human immunodeficiency virus (HIV) infection without related clinical issues, and 49 reported a previous hepatitis infection without complications. The Institutional Review Board of Azienda Ospedaliero Universitaria Careggi accepted the proposal of this retrospective study, and all selected patients were properly informed before surgery about the treatment and follow-up visits after discharge (cod. DCMT/ort12-m2-y01; date 12 February 2001).

### 2.1. Surigical Technique

All patients were operated on by two surgeons over the years, with the same surgical technique, the same implant (Genesis II^®^, Smith & Nephew, Indianapolis, IN, USA), general anaesthesia, short-term antibiotic prophylaxis (preoperatively: 1 g of endovenus vancomycin and 2 g of endovenous cefazolin; postoperatively 1 g of ev vancomycin every 12 h and 1 g of ev cefazolin every 8 h for 24 h. In the case of B-lattamic allergy, only the vancomycin was administered; in the case of tailored haematological prophylaxis, depending on the type of haemophilia affecting the patients, infusive boli was provided 30 min before anaesthesia. A pneumatic tourniquet applied at the level of the upper thigh and inflated to about 250 mm Hg was used in all cases. The tourniquet was released after the cement had set, allowing haemostasis before wound closure.

A standard longitudinal midline incision with a medial parapatellar approach was used in all patients with no previous surgery. When present, previous scars were utilized with the normal rules adopted for total knee revision arthroplasties. An extensive removal of the anterior, posterior, lateral, and medial synovial membrane was performed. Anterior and posterior bone surfaces were left untouched in order to maintain stability in flexion. The Genesis II (Smith & Nephew, Indianapolis, IN, USA) total knee arthroplasty used is a modular implant whose main features are the presence of an asymmetrical tibial base plate to match the cut surface of the tibia and a femoral component to ensure flexion space filling without external rotation. The femoral component was specifically designed with a thicker postero-lateral femoral condyle compared to the postero-medial femoral condyle. The trochlear groove was designed to allow patellar tracking in a more anatomical manner.

All patients underwent the same rehabilitative protocol in the same facility and were discharged with planned periodic evaluations at the outpatient clinic (1, 6, 12 months; every year postoperatively for the following 5 years; then, every 3 years).

### 2.2. Clinical and Radiographic Evaluations

All patients underwent a clinical evaluation for pain (Visual Analogue Scale—VAS); function (Haemophilia Joint Health Score—HJHS; Knee Society Score—KSS) [35,36]; Forgotten Joint Score-12 (FJS-12) for joint awareness during activities of daily living [37]; Western Ontario and McMaster Universities Osteoarthritis Index (WOMAC osteoarthritis index) for a self-reported outcome measure about pain, stiffness and pain [38]; and Range of Motion (ROM) of the operated joint, as well as a standard radiologic study (weight-bearing full leg X-rays, weight-bearing antero-posterior and lateral views), to assess the severity of the arthropathy following the Pettersson score [39].

The Knee Society Rating System was used for patient evaluation. Two separate scores were assigned: one for walking, stair climbing, and the use of walking aids (functional score), and another for pain, range of motion, and stability (knee score). Knee scores greater than 90 points were considered as excellent, 80–89 as good, 70–79 as fair, and less than 69 as poor. The FJS-12 is composed of 12 items, measuring the patient’s ability to forget the presence of an artificial joint in their daily life. For each item, there is a five-point Likert scale response. The raw results are converted to a 0–100-point scale. The highest score corresponds to a good outcome with the patient not being aware of the presence of the prosthesis [40]. The WOMAC score is used to determine the improvement following knee arthroplasty (KA). Its items are pain, stiffness, and physical function. The final score is from 12 to 96. [41,42].

VAS, HJHS, KSS, FSJ-12, and WOMAC scores were recorded at every follow-up visit, in particular at 1, 3, 6, 12, 24, and 48 months and then every 3–5 years after surgery.

Standard antero-posterior and lateral views weight-bearing X-rays were performed at 1, 12, and 48 months postoperatively, and then every 3–5 years.

The radiolucency results were documented in millimetres by the zone of the prosthesis in both the coronal and sagittal planes for the femur and tibia according to the method recommended by The Knee Society [43].

The frequency of hemarthrosis before and after knee replacement was documented.

### 2.3. Statistical Analysis

The statistical analysis was performed using the SPSS^®^ statistics software (version 23.0 for Windows, IBM Corporation, Armonk, NY, USA).

Statistical analysis was first performed based on an a priori assumption of *p* = 0.05 and calculation of variance to justify that the population from which it was extracted was generally homogeneous. All data were tested for normal distribution using the Kolmogorov–Smirnov test. Finally, the Student’s *t*-test was used both to compare preoperative and postoperative clinical scores. The non-parametric Kaplan–Meier estimator with 95% of confidence intervals was calculated using the Rothman formula; aseptic and septic loosening or instability requiring revision surgery were the endpoints. Survival tables were constructed using 12-month intervals. For each interval, the total number of TKAs entering the interval, the number of failures and withdrawals, the number at risk, the annual rates of failure and success, and the cumulative success rate were calculated.

## 3. Results

The mean follow-up was 12.3 years (range: 4.2–20.6 years). The mean Pettersson score at the time of surgery was 11.6 (range: 10–13). No early failures, no infections, and no intra-operative complications were documented. Three patients deceased during the years for clinical issues not related to TKA (11, 16, and 20 years after surgery, respectively). The mean surgical time was 68.4 min (range: 50–119). Only two patients needed blood transfusions (not related to vascular damage). All patients were able to begin the rehabilitation protocol starting within two days after surgery and performed continuous passive mobilization and weight-bearing exercises before discharge to the rehabilitative unit. At the time of discharge from the hospital, all patients were able to walk with full weight bearing, with two crutches. All clinical and functional scores improved significantly from pre-operation to the latest follow-up (*p* < 0.05). The mean preoperative HJHS score was 13.4 (range: 9–22); postoperatively, at the last follow-up, the mean score was 1.9 (range: 1–5). VAS scores improved from a mean preoperative value of 6.9 (range: 5–9) to a mean postoperative value of 1.3 (range: 0–3) at the latest follow-up. The mean preoperative ROM was 42.4° (range: 15–85°), and the mean post-operative value was 83.6° (range: 55–115°). Similarly, KSS scores improved from a mean value of 19.4 (range: 14–36) to a mean final score of 79.0 (range: 68–92). The WOMAC score improved from a mean value of 51.4 (range: 33–62) to a mean final score of 77.0 (range: 59–89). The FJS-12 score improved from a mean value of 49.3 (range: 35–59) to a mean final score of 61.0 (range: 53–81). Symptoms and functional impairments were improved in all cases, and most of the patients reported satisfaction with excellent outcomes. However, given the involvement of other target joints (mostly ipsilateral ankle and contralateral knee and/or ankle, one or both elbows) affecting the postoperative recovery, in 12 cases, the operated subjects referred to their outcomes rather as good.

**Figure 2:**
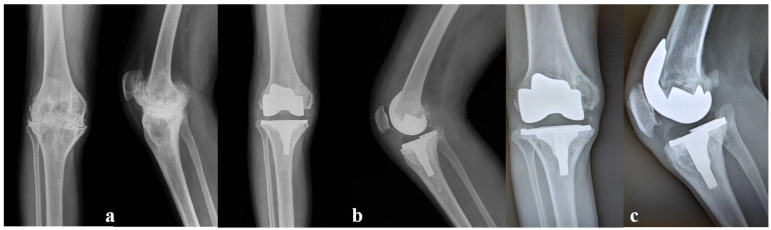
A 39-year-old patient with severe arthropathy of his left knee—Pettersson score 12 (**a**), undergoing a posterior stabilized TKA. X-rays at 1 year (**b**) and 16 years (**c**) after surgery. The implant was still in place without significant osteolysis or radiolucency. The patient was still satisfied, and his joint was well functioning.

**Figure 3:**
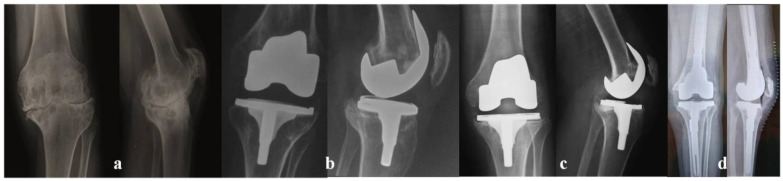
A 34-year-old and heavy worker patient with severe arthropathy of his right knee—Pettersson score 11 (**a**), undergoing a posterior stabilized TKA. X-rays at 1 year (**b**) and 13 years (**c**) after surgery. A symptomatic aseptic loosening was demonstrated, and the patient underwent a revision with an oxidized zirconium femoral component, titanium tibial component, and posterior stabilized high-flexion PE insert with cementless stems (**d**).

From a radiological point of view, the preoperative alignment was, in all cases, in varus deviation, with a mean angle of 12.1° (range: 1–19°), reaching a mean value of 4.3° in valgus deviation (range: 0–7°) (Figure 2). No periarticular ossifications were found, and in the first 4 years postoperatively, no radiolucency or osteolysis was recorded. In 15 patients, radiolucency was observed with a low progression over time, and one case of osteolysis was found. There was a progression of these alterations in only three patients. In one patient with severe haemophilia A and inhibitors (alloantibodies against coagulative factors used for treatment), 4 years after the index operation, due to recurrent bleedings, an early mechanical loosening of both components was recorded. The patient underwent a revision with cementless stems and a higher constraint implant. In another patient, a heavy worker with severe haemophilia A, 13 years after TKA, a femoral aseptic loosening was recorded. The patient underwent a revision with an oxidized zirconium femoral component and a constrained implant with cementless stems and wedges (Figure 3). A third patient, 16 years after surgery, showed progressive radiolucency but without any mechanical symptoms, and he had still not been scheduled for surgery at the latest follow-up.

**Figure 4:**
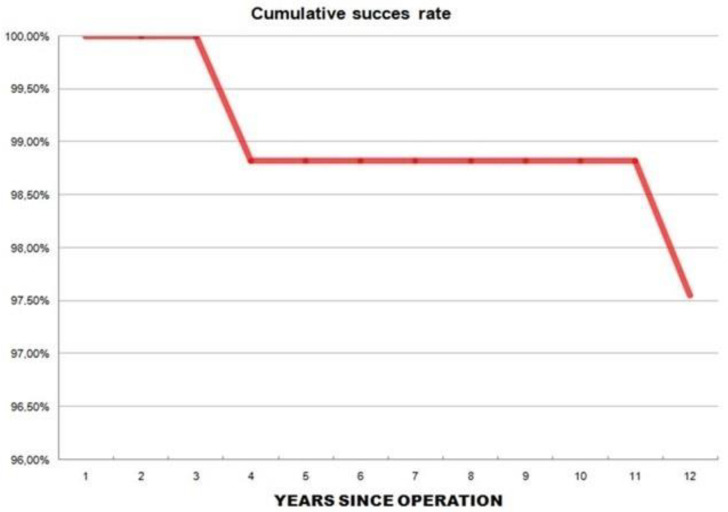
The Kaplan–Meier curve shows very good survival rates of the implants.

The overall survivorship of the implants was 97.5% at the latest follow-up (Figure 4).

## 4. Discussion

Haemophilia is a haemorrhagic disease inducing damage to the joint and its structures, leading to a status of chronic synovitis and then severe arthropathy [1,8]. The result is a complete disruption of the shape and function of the involved joint in very young subjects. Therefore, TKA in such patients is often challenging due to a severely altered anatomy and bony deformity, bony defects, high levels of soft tissue contracture, and muscle atrophy, often leading to high rates of postoperative complications. However, TKA has shown positive outcomes when performed in dedicated centres, despite the higher rates of complications with respect to TKA performed for primary osteoarthritis [18,34,44]. Nonetheless, functional outcomes and survival rates of TKA in such patients have been generally reported as inferior to osteoarthritic patients. The majority of experiences reported in the literature involved the use of standard chrome-cobalt femoral components and titanium tibial plates; these materials are the same as those usually chosen for elderly patients worldwide. As mentioned, PWH typically need TKA at a young or adult age, when the choice of better-performing implants should be made. To date, the longest follow-up reported using conventional cr-co prosthesis was shown by Ernstbrunner et al. The authors reported 18 years (SD ± 4) of survivorship of 15 patients (21 knees) out of 30 consecutive patients (43 knees) undergoing TKA due to haemophilic arthropathy. In 13 (30%) of the 43 consecutive knees, revision surgery was necessary due to infection or aseptic loosening, among which eight (19%) occurred due to aseptic loosening and five (12%) occurred due to haematogenous infection. The 15- and 20-year survival rates were 78% and 59%, respectively. Moreover, the authors reported that all patients with the primary TKA in situ at the latest follow-up observed progressive radiolucent lines around the implants [45]. More recently, Song et al. described a 10-year survival rate of 97% using standard implants in 131 knees. The mid-term results of TKA in haemophilic arthropathy were satisfactory in pain relief, improved function, and decreased flexion contracture. The authors remarked the fact that bleeding and PJI continued to be major concerns for TKA in haemophilic arthropathy, and the risk of periprosthetic fracture should be always taken into account for patient education and appropriate prevention [46].

Only few experiences have been reported in the literature concerning materials with in vitro high-performing tribological properties compared to the standard ones [47,48,49,50]. Such series have mainly focused on the use of oxidized zirconium components for younger patients, as well as on metal hypersensitive subjects with survival rates ranging from 100–98.7% at 5–7 years to 97.8% at 10 years [51,52]. Oxidized zirconium is composed of Zr (97.5%) and niobium (2.5%). It is produced by submitting the alloy to heat in air to greater than 500 °C. Thermal oxidation occurs, and as the oxygen diffuses through the alloy, the immediate surface oxidizes into a Zr ceramic approximately 5 lm thick. The alloy immediately underlying the ceramic surface has a high oxygen concentration, and this gradually decreases until the alloy is just composed of the two base materials. This does not result in a coated surface treatment, but rather in a gradual transition of the material and its properties; the finished product is a stable monolithic crystalline structure. Thanks to those properties, the oxidized zirconium was one of the best-performing materials introduced during the last decades for the following rationale: the younger the patient, the lesser wear should be obtained, and the longer the survivorship has to be expected [47,53,54]. The reason for such use can be attributed to fact that the oxidized zirconium femoral component for TKA has shown promising results in some laboratory analyses, with better wear properties than CoCr when articulating with ultra-high molecular weight polyethylene causing a reduction of PE wear and secondary osteolysis and an improvement in long-term survival of knee joint arthroplasties [47,53,54].

The first report on the adoption of this material in haemorrhagic patients was proposed by Innocenti et al., obtaining very good outcomes and no failure at short- to mid-term follow-up in haemophilic subjects. The authors reported that at the final follow-up, the knee score improved from an average of 23 points (11 to 45) to 86 points (62 to 100; *p* < 0.001), the mean knee flexion contracture improved from 22 degrees (0 degrees to 45 degrees) to 3 degrees (0 degrees to 10 degrees; *p* < 0.0001), and the mean total flexion arc improved from 69 degrees (5 degrees to 130 degrees) to 92 degrees (80 degrees to 145 degrees *p* < 0.001) [55]. Later, Carulli et al. proposed a series of primary and revision TKA with the same modular implant but with a long-term follow-up. The authors reported a single failure (aseptic loosening) in a series with a median follow-up of 12.2 years (3–21) for a group of primary TKA, and 8.6 years (4–12) for a group of revision TKA with an overall survival rate of 94.7% at 15 years [56]. No other similar experiences have been reported. The most probable cause of this limited series is related to the high costs of an implant made with such materials [57]. However, in the authors’ experience, higher costs are widely justified when performing a joint replacement in very young patients with a long life expectation, undergoing surgery with more consistent costs related to the recombinant coagulative prophylaxis [58]. In the present study, we reported a 97.5% survival rate at 12-year with just three failures.

In our series, the mean postoperative hip-knee alignment was 4°. This result is related to the severe preoperative deformities and very low quality or defect of bone, both on the femoral and tibial sides. Obtaining a pure mechanical alignment using a modern implant with highly performing material and adopting the best available haematological prophylaxis, we obtained a high survival rate. It would be interesting to evaluate the outcomes of robotic-assisted TKA as reported by Song et al. in the future. In their series, the postoperative axis was mechanically neutral at 0° [46].

The present study has some limitations. The study population was not highly consistent, as haemophilia is a rare condition; however, it represents the most unique series to date reported with the use of a specific modern implant for a long-term period. No control group was considered since, from the beginning of our experience with the surgical treatment of PWH we, decided to adopt the single best-performing implant from a tribological point of view.

## 5. Conclusions

Knee arthroplasty in haemophilic patients is still the most performed surgery despite improvements related to modern haematological prophylaxis. This surgery has a high rate of success, but survivorship is still debated. One of the factors that the history of joint replacement has demonstrated is the quality of prosthetic materials and their tribology. The present series showed a high survival rate of the implants, surely due also to this choice of oxidized zirconium coupled with highly crosslinked PE. Thus, we promote the use of modern implants in these patients in order to ensure long-lasting positive outcomes.

## Data Availability

All data are available in clinical records of the hospital (on paper up to 2016, on electornic format from 2017).
